# Healthcare professionals’ knowledge and attitudes on termination of pregnancy in eThekwini

**DOI:** 10.4102/safp.v67i1.6005

**Published:** 2025-02-12

**Authors:** Tashnee Singh, Kantharuben Naidoo

**Affiliations:** 1 Department of Family Medicine, Faculty of Family Medicine, University of KwaZulu-Natal, Durban, South Africa

**Keywords:** choice on termination of pregnancy, termination of pregnancy, knowledge, attitudes and practices regarding termination of pregnancy, TOP and healthcare, South Africa

## Abstract

**Background:**

While the *Choice on Termination of Pregnancy* (CTOP) *Act No. 92* was legalised in 1996 permitting termination of pregnancy (TOP) to be accessed at various public health facilities in South Africa, unsafe abortions continue to take place outside of legally designated facilities. The aim of this study was to explore the knowledge, attitudes and practices of healthcare professionals regarding TOP services at public primary healthcare (PHC) centres in the central business district (CBD) of the eThekwini Municipality in KwaZulu-Natal Province, South Africa.

**Methods:**

This quantitative, descriptive analysis used a questionnaire to obtain data from the healthcare professionals on four areas: demographic details, knowledge of (8 questions), attitudes to (10 questions) and practices (10 questions) regarding TOP services.

**Results:**

Among the 91 participants (whose ages ranged from 32 to 48 years), 91.2% were female, 93.4% belonged to the Christian faith, 42.9% were professional nurses and 74.8% had more than 5 years’ work experience. The mean of the overall knowledge, attitude and practice score was 51.7%, 67.4% and 62.8%, respectively, which indicated poor knowledge, moderate attitude and unacceptable practice.

**Conclusion:**

While some healthcare professionals’ knowledge regarding TOP services was poor, their personal beliefs may have contributed to their attitudes and practices regarding whether to assist their patients to access such services, despite it being legal.

**Contribution:**

This study provides insight into the health worker barriers that impede TOP service provision and access in the CBD of eThekwini.

## Introduction

The *Choice on Termination of Pregnancy* (CTOP) *Act No. 92*^1^ was legalised in 1996 during South Africa’s democratic transition, before which termination of pregnancy (TOP) was only permitted legally in cases where the pregnancy constituted a threat to the life of the mother.^2^ In spite of the earlier prohibition, women from all population groups sought to terminate unwanted pregnancies, despite the dangerous complications of infection, sterility and death, as well as risking exposure and criminal prosecution. All this was supposed to change with the *CTOP Act* (no 92 of 1996), when TOP could be legally accessed at various public health facilities, negating the need for illegal procedures. This Act allows for TOP on request up to and including 12 weeks of gestation, and in cases of socio-economic hardship, rape, incest and for reasons related to the health of the pregnant woman or foetus, termination can be performed up to 20 weeks. Thereafter, it is only available if the pregnancy causes severe threat to the life of the mother or if severe foetal congenital abnormalities exist.

The latest Saving Mothers Report 2020–2022 notes that unsafe abortions were still an avoidable factor in 16% of the deaths because of miscarriage.^3^ This suggests that a significant number of women continue to choose unsafe abortion outside of legally designated facilities in South Africa. The main reasons found for not using TOP services were not knowing that such a law exists, its provisions (54%) or how to access them (15%), anticipating health worker rudeness (17%), afraid of being found out (7%), too late in the pregnancy or a long waiting list (7%).^4^ These findings indicate that a lack of knowledge about abortion rights and perceived concern about TOP clinic staff attitudes were the most important barriers to safe and legal TOP.

Globally, other studies among healthcare professionals showed that knowledge of TOP legislation was poor with up to 88% not having received training on TOP services.^5,6,7^ Furthermore, despite safe TOP reducing maternal morbidity and mortality, the procedure continues to carry much of the stigma that leads to negative attitudes and contributes towards not referring women to dedicated facilities that offer such care.^5^ Nationally, it has been found that healthcare professionals who work in TOP centres have good knowledge of the *CTOP Act*.^8,9^ Additionally, TOP still carries stigma and may cause conflict in healthcare providers whose personal religious or cultural norms override their professional requirements to provide supportive services to everyone, including those who have decided to receive TOP service.^8,9,10^ Absence of training on the *CTOP Act* and a lack of support from managers were a consistent finding with up to 84% of professional nurses not receiving training to provide TOP services.^9,10^ Regionally, there is little information about the knowledge, attitudes and practices of healthcare professionals on TOP services.

Healthcare professionals have a key role to play as providers of TOP services, by ensuring safe and confidential access to this service, and as educators and providers of reliable sources of information to patients.^6^ However, those who have poor knowledge and harbour negative attitudes towards TOP are unlikely to execute the appropriate legal practices required to those seeking access to such services. This emphasises the need to upskill healthcare workers at all levels of care to ensure that they enable women to access TOP and to identify barriers faced by healthcare workers to provide such services. In KwaZulu-Natal Province and in South Africa, little is known about the knowledge, attitudes and practices among primary healthcare (PHC) workers regarding TOP service provision. This is important as it relates to facilitating referral to appropriate care.

The central business district (CBD) of the eThekwini Municipality, the port city of Durban, not only provides employment for many women but is home to many people who do not have the means to access private healthcare. Clinics in the CBD therefore offer services to women who rely on the staff having the appropriate knowledge to assist them in accessing various services, including TOP. This makes it essential for the healthcare professionals to be knowledgeable about the TOP services to ensure that those who rely on public sector services can access the care they need, even if they cannot pay for it. In the absence of such services, women may turn to illegal terminations of pregnancy should they fall pregnant and not want to keep the baby, thereby posing serious risks to their health and safety.

The aim of this study was therefore to explore the knowledge, attitudes and practices of healthcare professionals regarding TOP services at public PHC centres in the CBD of the eThekwini Municipality in KwaZulu-Natal province, South Africa. The results could identify health workers’ barriers to providing such services and facilitate improvements in their knowledge, attitudes and practices, giving them and their patients the confidence to seek care at these clinics.

## Research methods and design

This descriptive study was conducted at four PHC clinics, one hospital gateway PHC clinic and one community health centre (CHC) in the CBD of Durban, eThekwini Municipality. These six facilities do not perform TOP services onsite but refer all patients (with a referral letter) to the regional referral hospital TOP clinic, which is open every weekday. The study was a quantitative, descriptive analysis to establish healthcare professionals’ knowledge, attitudes and practices regarding TOP service provision using a questionnaire. The CBD encompasses a total of six clinics, and all six clinics were chosen to be part of this study. The target population included all healthcare professionals working at these respective clinics, who provided advice to patients such as counsellors, enrolled and professional nurses, nurse-midwives and doctors. The study was conducted from 01 February 2021 to 11 February 2021.

Every healthcare professional present at the respective clinics was invited to participate, equating to a total sample population of 100. A statistician assisted in determining the number of participants required to power this study. A sample size of 85 was required to estimate the proportion of healthcare professionals with good knowledge about TOP to within ± 15%, assuming a baseline of 50% participation. If the non-response or refusal rate was 10%, then a target of 94 was required. Stata version 13 statistical software was used to calculate the sample size.

The structured questionnaire was designed based on a literature review using the Mesh terms for search: ‘knowledge, attitudes, and practices of healthcare workers on TOP’. To improve content validity, two South African family physicians were invited to provide input into the tool prior to commencement. The questionnaire consisted of four sections: demographic data (age, gender, religion, category of provider and number of years of practice), healthcare professionals’ knowledge of, attitudes to and practices regarding TOP services. Knowledge was tested with four single best answer questions and four questions based on a Likert scale, where agree and strongly agree were regarded as correct. This was then expressed as a percentage of the total responses. Attitude consisted of 10 questions and practice of 10, each using a 5-point Likert scale, where 1 = strongly disagree and 5 = strongly agree, total scores being summed. To pilot the questionnaire for content, clarity and ease of understanding, a focused group pilot was held at Wentworth Hospital, a district hospital that provides TOP referral services, with 10 healthcare workers who work in the women’s health department. The questionnaire was tested and amended in response to feedback received, with participants completing it in a separate room, and the principal investigator (PI) being available to answer any questions.

### Data analysis

The data were entered into an Excel spreadsheet and analysed, with the Likert scores on the negatively worded questions being reverse-scored. Healthcare professionals’ knowledge, attitude and practice levels were defined as ‘good’ or ‘poor’ based on Bloom’s cut-off point. Healthcare professionals with knowledge scores above 60% were regarded as having good knowledge, while those with scores below 60% were considered having poor knowledge. Healthcare professionals with attitude scores of 80% and above were considered as having a good attitude, while those within the range of 60% – 79% moderate and score below 59% were regarded as having an unacceptable attitude. For practice section, healthcare professionals with scores > 80% and < 80% were classified as having acceptable and unacceptable practices, respectively.

Stata version 13.1 was used for the statistical analysis, with descriptive statistics being used to summarise the data. Demographic characteristics were presented as percentages. Knowledge data was presented as percentage correct. Attitude and practice data were presented using a Likert categorical scale. The frequency distribution was checked for normality, and means or medians were reported as appropriate. Subgroup comparisons of scores by number of years of practice, age group or category of provider were done using analyses of variance (ANOVA) or Kruskal-Wallis tests.

### Ethical considerations

Ethical clearance to conduct this study was obtained from the University of KwaZulu-Natal, Biomedical Research Ethics Committee (No. BREC/00001078/2020). Permission obtained to undertake the study by the KwaZulu-Natal Department of Health and the eThekwini Municipality Health Department Research Committee and individual site approval were granted by the six public PHC clinics. Written informed consent was obtained for every questionnaire administered, with participants being allocated codes to ensure confidentiality and anonymity. The completed questionnaires were stored in a lock-up facility with password encryption only accessible to the PI for the digital data.

## Results

### Demographic characteristics

Of the 100 who were invited to participate, 91 completed the questionnaire. Most (*n* = 81, 89.1%) participants were working at PHC clinics, and the remainder (*n* = 10, 11%) in a CHC. (Table 1). The median age was 38 years old, with an interquartile range (IQR) of 32–48. The majority (*n* = 83, 91.2%) were female, with most being of the Christian faith (*n* = 85, 93.4%). Most were professional nurses (*n* = 39, 42.9%), with three-quarters (*n* = 68, 74.8%) having more than 5 years of work experience.

**TABLE 1:** Demographic characteristics of study population.

Variable	Characteristic	*n*	%	Median	IQR
Clinic type	PHC Clinic A	20	22.0	-	-
	PHC Clinic B	9	9.9	-	-
	PHC Clinic C	21	23.1	-	-
	Hospital Gateway PHC Clinic	20	22.0	-	-
	PHC Clinic D	11	12.1	-	-
	CHC	10	11.0	-	-
Age group (years)		-	-	38	32–48
	< 30	14	15.4	-	-
	30–39	34	37.4	-	-
	40–49	24	26.4	-	-
	50–64	19	20.9	-	-
Gender	Male	8	8.8	-	-
	Female	83	91.2	-	-
Religious affiliation	Christian	85	93.4	-	-
	Hindu	3	3.3	-	-
	Muslim	1	1.1	-	-
	Other	2	2.2	-	-
Category of provider	Counsellor	12	13.2	-	-
	Enrolled nurse	27	29.7	-	-
	Professional nurse	39	42.9	-	-
	Nurse-midwife	8	8.8	-	-
	Doctor	3	3.3	-	-
	Other (Allied health professionals)	2	2.2	-	-
Years of practice		-	-	10	5–15
	≤ 5	23	25.3	-	-
	6–10	27	29.7	-	-
	> 10	41	45.1	-	-

PHC, primary healthcare; CHC, community health centre; IQR, interquartile range.

### Knowledge

Knowledge was assessed by the percentage of correct responses to questions. These questions related to issues of consent, confidentiality and provisions under the *CTOP Act* (Table 2). Issues of consent related to age and disclosure to a third party, such as parents in the case of a minor that is a patient under the age of 18 years. Less than half (*n* = 42, 46.2%) know that no consent other than that of the pregnant woman, even in the case of a minor, is required for a TOP. Issues of confidentiality indicated that three-quarters (*n* = 68, 74.7%) know that the identity of a woman who has requested a TOP shall always remain confidential unless she herself chooses to disclose that information. Three questions related to the provisions of law regarding TOPs from 13 to 20 weeks, with one-third (*n* = 30, 33.0%) knowing that TOP is available for poor socio-economic status (*n* = 73, 80.2%), knowing that TOP is performed in the case of rape (*n* = 28, 30.8%), and knowing that TOP is available when it comes to incest but with gestational limits. Half (*n* = 48, 52.7%) know the provisions of law above 20 weeks of gestation and that counselling and psychological services are required in the case where pregnancy resulted from rape. Among the respondents, 59.4% (*n* = 54) know that above 20 weeks of gestation TOP is available if severe foetal congenital problems are present. Less than half (*n* = 33, 36.3%) know that poor socio-economic status is an indication to have a TOP but with gestational limits. Four of the eight knowledge questions received correct scores of over 50%, of which two were over 70%, the mean knowledge score being 51.7%.

**TABLE 2:** Application of knowledge questions (*N* = 91).

Number	Topic	Application of knowledge questions tested	Number correct (*n*)	Correct answer (%)
1	Issues of consent	No consent other than that of the pregnant woman, even in the case of a minor, is required for a TOP	42	46.2
2	Provisions under the law	TOPs between 13 and 20 weeks of gestation and women’s socio-economic status	30	33.0
3	Issues of confidentiality	The identity of a woman who has requested a TOP shall always remain confidential unless she herself chooses to disclose that information	68	74.7
4	Provisions under the law	TOPs above 20 weeks of gestation and patient’s additional social problems	48	52.7
5		TOPs and foetal congenital problems	54	59.4
6		TOPs between 13 and 20 weeks of gestation and rape	73	80.2
7		TOPs between 13 and 20 weeks of gestation and incest	28	30.8
8		TOPs and a woman’s socio-economic status	33	36.3
-	-	Mean score	47	51.7

TOP, termination of pregnancy.

### Attitude

Attitudes were explored with statements viewing TOP in a positive way, agreeing with having a TOP in certain situations and having unfavourable attitudes towards TOP (Table 3). Among respondents (*n* = 76), 83.5% were willing to refer women for TOP. Most (*n* = 60, 65.9%) agreed that women who have had previous TOPs should have access to these services for future unwanted pregnancies, with less than half (*n* = 44, 48.4%) disagreeing that their religion influenced their attitude to TOP. Three-quarters (*n* = 61, 67.1%) felt that healthcare professionals should be allowed to work in a TOP service as a choice and not as an obligation. On the matter of TOP for social reasons, one-third (*n* = 30, 33.0%) agreed that it is unfair, while most (*n* = 57, 62.7%) agreed that it should not be performed in a patient with a gestation ≥ 20/40 weeks. Half (*n* = 48, 52.8%) agreed that healthcare professionals should be penalised for obstructing access to TOP services. With respect to the issue of perceived staff being caring, three-quarters were either neutral or agreed, while a majority (*n* = 63, 69.3%) disagreed that these staff were unfriendly. Most participants (*n* = 55, 60.5%) indicated that they disagreed that their attitudes towards TOP are influenced by those of their community. The group mean score for attitude was 33.7 out of 50 points (67.4%), with a standard deviation (s.d.) of 4.9 (9.8%), and minimum and maximum scores of 21 (42.0%) and 45 (90.0%), respectively.

**TABLE 3:** Likert questions on attitude.

Number	Question	Strongly disagree		Disagree		Uncertain		Agree		Strongly agree	
		*n*	%	*n*	%	*n*	%	*n*	%	*n*	%
1	I personally have no problem referring a patient who requests a TOP	3	3.3	9	9.9	3	3.3	48	52.7	28	30.8
2	Women who have had previous TOPs should have access to these services for future unwanted pregnancies	7	7.7	15	16.5	9	9.9	49	53.8	11	12.1
3	My religious beliefs do not support me referring a patient for a TOP	17	18.7	27	29.7	9	9.9	22	24.2	16	17.6
4	Healthcare professionals should be allowed to work in a TOP service out of choice	10	11.0	15	16.5	5	5.5	29	31.9	32	35.2
5	TOP for social reasons is unfair	8	8.8	39	42.9	14	15.4	18	19.8	12	13.2
6	No TOP should be performed in a patient with a gestation ≥ 20/40 weeks	5	5.5	18	19.8	11	12.1	32	35.2	25	27.5
7	Healthcare professionals should be penalised for obstructing access to TOP services	17	18.7	16	17.6	10	11.0	36	39.6	12	13.2
8	Healthcare professionals who render TOP services are generally caring people	7	7.7	10	11.0	31	34.1	36	39.6	7	7.7
9	Healthcare professionals who render TOP services are unfriendly	33	36.3	30	33.0	23	25.3	2	2.2	3	3.3
10	The attitude of my community influences my attitude towards TOP	23	25.3	32	35.2	15	16.5	18	19.8	3	3.3

TOP, termination of pregnancy.

### Practice

In this study, two-thirds (*n* = 58, 63.8%) of participants have not been trained on the *CTOP Act*; most (*n* = 72, 79.1%) agreed that they refer all patients requesting TOP to the local service. Regarding providing pre-termination counselling to all patients requesting TOP, half (*n* = 49, 53.9%) agreed they do this. More than half (*n* = 51, 56.1%) were not aware or were uncertain of all the TOP services available in the sub-district or how to access them. Half (*n* = 46, 50.6%) agreed that they engage in discussions regarding additional social problems that patients requesting TOP may have. Half (*n* = 47, 51.7%) agreed the referral system for patients needing TOP functions well. Two-thirds (*n* = 59, 64.9%) were uncertain and did not feel supported by the district management team on giving adequate information and help to women undergoing a TOP. Most (*n* = 73, 80.3%) disagreed in that they do not receive regular in-service training on the *CTOP Act* and the services offered in the sub-district. Three-quarters (*n* = 70, 77.0%) agreed they will be very happy to receive regular updates on the *CTOP Act*. Half (*n* = 53, 58.3%) agreed they feel confident in referring patients to the TOP services. One-third (*n* = 35, 38.5%) confirmed conclusively that a copy of the *CTOP Act* of 1996 was available in their facility. The group mean score for practice was 31.4 out of 50 points (62.8%), with a s.d. of 6.9 (13.8%), and minimum and maximum scores of 10 (20.0%) and 50 (100%), respectively. A summary of the Likert questions for assessing practice can be found in Table 4.

**TABLE 4:** Likert questions on practice.

Number	Question	Strongly disagree		Disagree		Uncertain		Agree		Strongly agree	
		*n*	%	*n*	%	*n*	%	*n*	%	*n*	%
1	I have been trained on the *CTOP Act*.	25	27.5	33	36.3	5	5.5	21	23.1	7	7.7
2	I refer all patients requesting TOP to the local TOP service.	5	5.5	10	11.0	4	4.4	49	53.8	23	25.3
3	I provide pre-termination counselling for all patients requesting TOP.	9	9.9	20	22.0	13	14.3	33	36.3	16	17.6
4	I am aware of all the TOP services available in the sub-district and know how to access them.	8	8.8	26	28.6	17	18.7	28	30.8	12	13.2
5	I engage in discussions regarding additional social problems that patients who request TOP may have.	8	8.8	28	30.8	9	9.9	38	41.8	8	8.8
6	The referral system for patients needing TOP functions very well.	5	5.5	12	13.2	27	29.7	36	39.6	11	12.1
7	I feel supported by the district management team in providing advice to my patients on TOP services.	11	12.1	20	22.0	28	30.8	24	26.4	8	8.8
8	I receive regular in-service training on the *CTOP Act* and the services offered in the sub-district.	38	41.8	35	38.5	6	6.6	7	7.7	5	5.5
9	I would be very happy to receive regular updates on the *CTOP Act*.	5	5.5	10	11.0	6	6.6	44	48.4	26	28.6
10	I feel confident in referring patients to the TOP services.	6	6.6	14	15.4	18	19.8	42	46.2	11	12.1

TOP, termination of pregnancy; CTOP, choice on termination of pregnancy.

### Subgroup comparison of scores

On subgroup analysis of knowledge questions 1–4 (single best answer questions), the category of provider showed a statistical difference with regard to level of knowledge. In this study, religious affiliation was associated with differing attitudes towards TOP. Lastly, on subgroup analysis of practice, there was a statistical difference noted when looking at the category of provider. A summary of the subgroup analysis of knowledge, attitude and practice regarding TOP service can be found in Table 5. A *p*-value of < 0.05 was considered significant.

**TABLE 5:** Subgroup analysis of knowledge, attitude and practice regarding termination of pregnancy service.

Variable	*n*	Knowledge questions 1–4			Knowledge questions 5–8			Attitude			Practice		
		Mean	s.d.	*p*	Mean	s.d.	*p*	Mean	s.d.	*p*	Mean	s.d.	*p*
**Age group (years)**	-	-	-	0.90	-	-	0.90	-	-	0.40	-	-	0.52
< 30	14	2.00	0.96	-	13.43	3.30	-	35.57	5.24	-	29.00	7.81	-
30–39	34	2.09	1.00	-	12.71	2.32	-	33.21	4.89	-	31.47	7.86	-
40–49	24	2.00	1.14	-	12.92	2.55	-	33.88	4.92	-	31.88	5.50	-
50–64	19	2.16	1.17	-	13.11	2.33	-	32.79	4.71	-	32.47	6.03	-
**Gender**	-	-	-	0.80	-	-	0.60	-	-	0.44	-	-	0.30
Female	83	2.07	1.05	-	12.99	2.35	-	33.78	4.89	-	31.64	6.49	-
Male	8	2.00	1.20	-	12.63	4.14	-	32.38	5.29	-	29.00	10.58	-
**Religion**	-	-	-	0.21	-	-	0.40	-	-	0.02	-	-	0.40
Christian	85	2.02	1.03	-	12.87	2.41	-	33.35	4.92	-	31.24	7.02	-
Other	6	2.67	1.21	-	14.17	3.82	-	38.00	2.19	-	33.83	4.71	-
**Category of provider**	-	-	-	0.02	-	-	0.52	-	-	0.09	-	-	0.04
Counsellor	12	1.58	1.08	-	12.50	2.07	-	30.00	5.03	-	28.75	6.74	-
Enrolled nurse	27	1.67	1.04	-	12.44	2.67	-	34.67	5.03	-	32.00	2.00	-
Professional nurse	39	2.33	0.98	-	13.03	2.38	-	33.26	4.71	-	28.59	6.66	-
Nurse-midwife	8	3.13	0.35	-	14.88	3.36	-	34.67	4.77	-	33.77	6.58	-
Doctor	3	1.67	0.58	-	13.00	1.00	-	35.13	4.94	-	33.38	7.48	-
Other	2	1.50	0.71	-	13.50	2.12	-	34.00	2.83	-	30.50	6.36	-
**Years of practice**	-	-	-	0.34	-	-	0.46	-	-	0.28	-	-	0.69
≤ 5	23	1.83	0.83	-	12.48	2.56	-	33.96	5.07	-	31.70	6.90	-
6–10	27	2.26	1.16	-	13.00	2.86	-	34.70	4.68	-	30.44	8.89	-
> 10	41	2.07	1.08	-	13.20	2.28	-	32.80	4.95	-	31.88	5.36	-

s.d., standard deviation.

## Discussion

This study aimed to highlight the demographic characteristics, as well as the knowledge, attitudes and practices of 91 healthcare professionals regarding TOP service provision in six clinics in the CBD of Durban. Key findings were that the knowledge of healthcare professionals regarding TOP was poor, attitudes towards TOP services were moderate and practice was unacceptable.

### Knowledge

The poor overall knowledge score is concerning, considering most participants having more than 5 years of work experience. Those over 40 years possibly have worked for 20 years, during which time the provisions of the Act should have been implemented. One possible explanation is that only one-third have access to the *CTOP Act* in the facility where they work, and that in the absence of being reminded about its provisions, their patients’ rights have been forgotten. In a study conducted in Ethiopia, 81.1% of healthcare professionals had good knowledge regarding legislation on safe abortion care, with most having access to the abortion guidelines in their workplace.^11^ In another study conducted in South Africa, it was established that most nurses were knowledgeable about the *CTOP Act*, with 56% having a copy of the Act in the facility where they work.^9^

Of concern was the lack of knowledge that minors (aged 18 years and below) do not need parental consent for a TOP (46.2%), and the fact that 25% did not know that the identity of someone who has had a TOP should remain confidential, highlighting issues raised elsewhere about poor staff attitudes and confidentiality.^4^ While most (80.2%) knew that a TOP up to 20 weeks was allowed in the case of rape, only 59.4% know that it was permitted for foetal congenital problems above 20 weeks of gestation, and 30.8% for incest, raising concerns about their attitudes to women who seek care with such issues and their practices of treating them with kindness and referring them on to have a TOP during the allowed time. This is of concern as it may have the unintended consequence of causing women to then seek TOPs from illegal providers, putting them at a greater risk of morbidity and mortality.^12^ Ensuring that the healthcare professionals have good knowledge is essential for enabling them to help their patients make informed decisions and direct them to where they can access the services.

### Attitude

Termination of pregnancy is an extremely sensitive issue, being influenced by several cultural, religious and societal factors, with 16.5% indicating that they had a problem referring patients for a TOP on request. This suggests that while women have a legal right to a TOP, for any reasons, the healthcare professionals they encounter could not be open to directing them to such a service because of their own personal opinions, which are influenced by their religion and community. This is like other studies conducted in South Africa, which indicate that women’s rights to TOP cannot be assumed to occur when the people advising them may have different opinions and not be willing to compromise their own beliefs or attitudes for their patients. A South African study revealed that some healthcare workers’ religious and personal beliefs and the fear of being ostracised by their community played an important role in their decisions not to be involved in TOP services.^8^

Most participants (67.1%) felt that healthcare professionals should be allowed to work in a TOP service as a choice and not an obligation so as not to infringe on their personal or religious beliefs. Some healthcare workers may link it to their previous exposure to morbidity and mortality associated with illegal TOP, as reported elsewhere.^8,9^ The *CTOP Act* makes allowance for a health provider’s right to conscientiously object and refuse to perform a TOP, but they are obliged to inform women of their reproductive right to choose a TOP and refer them to another provider. As the included clinics were often the first point of contact for women seeking a TOP, all the staff should have been able to refer then onwards to secure such a service, irrespective of their experiences. Over half (52.8%) agreed that healthcare professionals should be penalised for obstructing access to TOP services. This is in keeping with the Act and ensures that women’s rights are protected, and that healthcare professionals’ preferences cannot be used to prevent women from accessing such services. Previous studies have reported that confusion with regard to healthcare professionals’ rights to conscientiously object to assisting women often interfered with TOP service provision.^8^

In this study, most participants (65.9%) agreed that women who have had previous TOPs should have access to these services for future unwanted pregnancies, this being linked to the concept of failed contraception and poor use of family planning measures. Studies conducted in South Africa indicated a bias against women who have had previous TOPs, as they were perceived as being irresponsible about using contraception and used the procedure as a contraceptive method.^8,9,10^

While there is limited research describing reasons why women seek TOPs, the literature indicates that poor socio-economic status is a main motivation in women seeking TOPs,^5^ as they cannot afford to have the child. The findings of this study are similar to those reported in Thailand, where participants were ambivalent about TOP for socio-economic reasons, which suggests an element of judgement about what constitutes legitimate reasons, despite abortion being an option on demand.^5^ This was not in keeping with the findings from another study conducted here in Cape Town, South Africa, which found healthcare professionals to be more sympathetic when it came to TOP for socio-economic reasons.^8^

According to the *CTOP Act*, after 20 weeks of gestation, TOPs are performed if the pregnancy causes severe threat to the life of the mother or if severe foetal congenital abnormalities exist. In this study, most participants (62.7%) agreed that no TOP should be performed in a patient with a gestation ≥ 20 weeks, which is not in keeping with the law. It has been reported to be because of healthcare professionals finding it too traumatic to deal with, as the foetus is then formed rather than being an embryonic sac, as reported elsewhere.^8,9^

In this study, less than half the respondents agreed that healthcare professionals who render TOP services were perceived as being caring, while a majority (69.3%) disagreed that these staff were unfriendly, both responses suggesting that they do not know about the way services are provided elsewhere in the TOP system. A previous study in South Africa found that more than one-third of the women who knew the *CTOP Act* were unwilling to access designated facilities because of anticipated staff rudeness.^4^ In another study carried out in South Africa, 56% of healthcare professionals agreed that nurses who rendered TOP services were generally caring people, while 88% disagreed with the statement that nurses who rendered TOP services were unfriendly.^9^

### Practice

The lack of training received by healthcare professionals on the *CTOP Act* may possibly explain their unacceptable responses regarding the associated services they provide. This further suggests that they either do not know their responsibilities or were intentionally denying women the right to access them, as prescribed in the Act. This absence of training is problematic, given the importance of their role in facilitating safe and effective care. In another study conducted in South Africa, 84% of professional nurses had not received training to provide TOP services.^9^

In a study that examined the counselling needs of women accessing services at a TOP clinic based in the same province in South Africa, it was found that clinic nurses had widely variable counselling training, their experience ranging from less than 2 months to 8 years.^13^ The pre-termination counselling process also did not accommodate for clients’ differing counselling needs, which includes requests for support from women experiencing intimate partner violence (IPV). Given the high rates of IPV in South Africa, healthcare workers must prepare for the likelihood that some patients who seek TOPs may be in violent relationships and engage in these conversations.

In this study, only 51.7% agreed that the referral system for patients needing TOP functions well, which means that the remainder either did not know or knew that it did not work well, both being concerning, as it could affect their chance of referring women to access it. This is consistent with a previous study, which found that health providers were concerned about the numerous difficulties women faced when seeking a TOP, such as a lack of adequate pre-termination counselling, disrespectful staff attitudes, overcrowded facilities and overburdened and fragmented service delivery.^8^ The findings for their feeling confident about referring patients for TOP services were similar to their previous responses, with 58.3% agreeing to this sentiment. Of concern with both responses is the high number of those who either disagreed or were uncertain, indicating the need for information about the referral process to be made available to all those involved with TOP services. Healthcare professionals need to have the confidence to assure women that they will be able to access the desired services, and how the systems and process work, as this will reassure them that they will get the care they need.

Two-thirds of the respondents (64.9%) were uncertain or did not feel supported by the district management team regarding giving them adequate information about helping women access a TOP, which indicates the need for managers to be aware of the gaps in their staff’s knowledge to enable it to be addressed if they are to provide the available services. This is similar to another study in South Africa, where healthcare professionals working in TOP services also did not perceive management to give much sufficient support.^9^ This may be so because of most (80.3%) not receiving regular in-service training on the *CTOP Act*, with many (77.0%) regarding regular updates and training as desirable. While the healthcare professionals may have relevant knowledge and supportive attitudes, the absence of management support may affect their ability to put into practice the aspects of the Act that they are required to do. Studies have recognised that TOP service provision is frequently reliant on a ‘core of committed providers’ and the lack of support, training and education can result in a reluctance of participation.^6^

### Subgroup comparison of scores

On subgroup analysis of knowledge questions 1–4 (single best answer questions), the category of provider showed a statistical difference with regard to level of knowledge. This has also been reported in a previous study where ‘medical’ job position compared to nursing or midwifery positively contributed to knowledge levels.^6^ In this same study, it was noted that male gender, educational level (diploma or certificate) and age less than 30 years old negatively contributed to overall knowledge, which was not the case in our study. Finally, both studies found the number of years of practice not to be linked to increased levels of knowledge, which suggests the need for comprehensive training and ongoing updates across all healthcare professionals irrespective of age or professional attributes.

Religious affiliation was associated with differing attitudes towards TOP in our study and a previous one conducted in Thailand,^5^ with healthcare professionals possibly experiencing moral conflict when they encounter situations that oppose their cultural or religious beliefs. Values clarification workshops were introduced in South Africa shortly after the implementation of the new *CTOP Act*, the intention being to establish the link between healthcare professionals’ knowledge, attitudes and personal belief systems and how this affected the practices related to TOP services. Healthcare professionals shared that values clarification helped them to view things with a new perspective and to define their role as facilitators within the TOP service who guide rather than direct their patients.^8^

## Conclusion

This study found that the knowledge of healthcare professionals in the busy CBD of the eThekwini Municipality regarding TOP was poor, which affected their attitudes to assisting their patients to access such services and therefore their practices. In many instances, their practices were not aligned to the *CTOP Act*, this being understandable, given their lack of knowledge and therefore their diverse attitudes towards its implementation. Women who attend health facilities to access TOP have made the decision not to seek illegal procedures and need to be supported and encouraged to access safe healthcare. Health worker barriers identified include issues such as a lack of confidentiality, overall moderate staff attitudes and healthcare professionals not being confident about referring women for TOP services, thus rendering such services inaccessible, which can result in women seeking care outside of the public health sector, putting their lives at risk.

### Recommendations

Although South Africa liberalised its abortion law in 1996, significant barriers still impede service provision and access, indicating the need to ensure that healthcare professionals are trained on the *CTOP Act* as a priority to ensure it can be appropriately implemented. Staff need to receive regular in-service training on the services offered in the sub-district, given that PHC clinics always refer to higher level institutions for more specialised care. The continuing success of the TOP service relies on the willing participation of staff and will be enabled by ensuring that they have the required knowledge to promote its uptake. Given that there is an Act that enables women to access TOP legally, irrespective of their own beliefs, healthcare professionals need to have positive attitudes towards enabling women to acquire the services they require, which will contribute to reducing the stigma associated with TOP for both them and their patients.

### Limitations

This study will not be able to be generalised outside the CBD of the eThekwini Municipality.
